# Oxidative Stress and Antioxidants in Pediatric Asthma’s Evolution and Management

**DOI:** 10.3390/antiox13111331

**Published:** 2024-10-31

**Authors:** Ileana Katerina Ioniuc, Ancuta Lupu, Felicia Dragan, Irina Tarnita, Monica Mihaela Alexoae, Violeta Streanga, Costica Mitrofan, Aye Aung Thet, Alin Horatiu Nedelcu, Delia Lidia Salaru, Stefan Lucian Burlea, Elena Cristina Mitrofan, Vasile Valeriu Lupu, Alice Nicoleta Azoicai

**Affiliations:** 1 Pediatrics, “Grigore T. Popa” University of Medicine and Pharmacy, 700115 Iasi, Romania; ileana.ioniuc@umfiasi.ro (I.K.I.); ancuta.ignat1@umfiasi.ro (A.L.); mihaela.alexoae@umfiasi.ro (M.M.A.); streangavioleta@yahoo.com (V.S.); vasile.lupu@umfiasi.ro (V.V.L.); alice.azoicai@umfiasi.ro (A.N.A.); 2 Faculty of Medicine and Pharmacy, University of Oradea, 410087 Oradea, Romania; 3 Faculty of Medicine, “Grigore T. Popa” University of Medicine and Pharmacy, 700115 Iasi, Romania; costel_mitrofan@yahoo.com (C.M.); ec5699l@gmail.com (A.A.T.); alin_nedelcu@yahoo.com (A.H.N.); deliasalaru@gmail.com (D.L.S.); 4 Public Health and Management Department, “Grigore T. Popa” University of Medicine and Pharmacy, 700115 Iasi, Romania; lucianburlea@yahoo.com; 5 CF Clinical Hospital, 700506 Iasi, Romania; criselend@yahoo.com

**Keywords:** children, bronchial asthma, antioxidants, oxidative stress

## Abstract

Within the pediatric population, bronchial asthma is one of the most prevalent chronic respiratory system diseases. The number of exacerbations, severity, and duration of symptoms all have a significant impact on children’s life quality. In the last decades, the prevention and management strategies of this pathology have focused on maintaining or even increasing the pulmonary function to maximum levels in early childhood, as it has been demonstrated that functional deficits at this level occurring before school age cause pathological manifestations later, in adulthood. The epithelium of the airways and implicitly that of the lung is the first barrier against the lesions caused by pro-oxidative factors. Both oxidative and antioxidative factors can be of endogenous origin (produced by the body) or exogenous (from the environment or diet). Good functioning of antioxidant defense mechanisms from the molecular level to the tissue level, and a balance between pro-oxidative factors and anti- oxidative factors, influence the occurrence of compensatory mechanisms at the level of the respiratory epithelium, causing the delay of local responses to the stress induced by chronic inflammation (bronchial remodeling, thickening of airway smooth muscles, bronchoconstriction, bronchial hyper-reactivity). These mechanisms underlie the pathophysiological changes in asthma. Numerous studies carried out among the pediatric population inclusively have demonstrated the effectiveness of antioxidants in the prophylaxis, slowing down and preventing the progression of this pathology. This review complements the scientific articles, aiming at emphasizing the complexity of oxidative physio-pathological pathways and their importance in the occurrence, development, and therapeutic response in asthma, providing a good understanding of the relationship between oxidative and antioxidative factors, and being a source of future therapeutic strategies.

## 1. Introduction

Bronchial asthma is one of the most common chronic airway pathologies, affecting approximately 5–20% of children worldwide. Asthmatic children are more prone to high rates of school absenteeism and limitations in physical activity [1].

The Global Initiative for Asthma (GINA) [2] defined this pathology as heterogeneous, in which the characteristic symptoms (wheezing, cough, and dyspnea), and their intensity, are constantly changing, along with variable bronchial obstruction during exhalation [3]. These symptoms can be triggered by factors such as physical exercise, exposure to allergens or bronchial irritants, climate changes, or viral respiratory infections. These symptoms can be caused by factors such as exposure to bronchial irritants or allergens, physical exercise, viral respiratory infections, or climate changes [4]. These triggering factors activate the structural cells of the airways and release approximately 100 pro-inflammatory mediators, including cytokines, chemokines, growth factors, lipid mediators, adhesion molecules, and inflammatory enzymes and inflammatory peptides [5,6]. These mediators recruit the cells of the immune system and, together, they make up a specific pattern of inflammation in patients with bronchial asthma characterized by mast cell degranulation, and the involvement of eosinophils and the T helper 2 (Th2) lymphocytes [7]. This inflammatory response is responsible for the subsequent clinical manifestations.

There are some diagnostic pitfalls in bronchial asthma in children, such as gastroesophageal reflux disease (GERD) and obesity. These two conditions are both significant risk factors that can complicate asthma in the pediatric population. Children with asthma are more prone to GERD, and it is estimated that a significant percentage of pediatric asthma patients also suffer from GERD, which can make asthma management more challenging [8,9,10,11,12]. Obesity is associated with a state of chronic low-grade inflammation, which can affect the lungs and airways, worsening asthma control in children [13]. A good management of these conditions could reduce the incidence of misdiagnosed bronchial asthma, as well as the number of factors that precipitate asthma symptoms in children.

Nowadays, the importance of maintaining or even increasing lung function to maximum values during childhood has been re-established by accumulating evidence showing that the functional deficits at this level installed before school age could also manifest in adulthood [14,15,16]. Therefore, obtaining an optimal lung function in childhood is an important goal in the prevention of chronic respiratory diseases [17]. The major features of asthma are chronic bronchial inflammation and bronchial hyper-reactivity, which causes recurrent obstruction of the airflow during exhalation. It is known that oxidative stress is inevitably present and contributes to the pathogenesis of inflammatory lung diseases, including bronchial asthma [6].

The tissues of the lungs, but especially the epithelium of the airways, are in direct contact with the oxidants in the ambient air and are susceptible to the action of reactive oxygen species (ROS), which cause inflammation and bronchial destruction. Although the complexity of the mechanisms that control the balance of antioxidative/oxidative compounds at the lung level is far from being fully understood, there is evidence that demonstrates that oxidative stress at the bronchial level is the result of an imbalance between the production of oxidants and the functioning status of the antioxidative systems, with the latter possibly being genetically modulated, thus obtaining different cellular responses [18,19].

Pulmonary matrix components (such as collagen and elastin) can be directly damaged by oxidants, causing fibrosis through phagocytic inhibition. This inhibition is associated with changes in IL6 and metalloproteinase 9 (MMP-9) production, both of which are key factors in airway remodeling and fibrosis [20,21].

Chronic inflammation of the central and peripheral airways in severe chronic asthma is also typically characterized by an increased number of activated T lymphocytes (particularly CD4+) [22]. Persistent chronic inflammation is known to contribute to parenchymal and branchial remodeling [23]. These processes of structural changes involve epithelial cell hyperplasia, reticular basement membrane thickening, collagen deposition, peri-bronchial fibrosis, the transition of the respiratory epithelium to the mesenchymal epithelium, and airway smooth muscle (ASM) hyperplasia [24]. Inflammatory reactions cause the destruction of the basal membrane by at least two mechanisms: the synthesis of proteases and the production of ROS. In subjects without lung damage, the intact basal membrane shows a dynamic balance between the synthesis and degradation of its components, mainly between proteases and anti-proteases. These enzymes are synthesized by mesenchymal cells such as fibroblasts, macrophages, endothelial and epithelial cells, and inflammatory cells [25].

The increased number of Th2 lymphocytes causes eosinophilic inflammation [26]. Interleukins have multiple functions in the inflammatory process as follows: IL-4 plays a role in Th2 differentiation and IgE synthesis [27], IL-5 is a key factor in eosinophil differentiation and survival, and IL-13 promotes hyperresponsiveness, bronchial mucus hypersecretion, eosinophilia, and airway remodeling [28].

The expression of inflammatory mediators is regulated by transcription genes which, in turn, are controlled by pro-inflammatory factors such as activator protein-1 (AP-1), signal transducers and transcription activators (STAT), and nuclear transcription factor (NF-kb) [29,30]. Some of these factors can be activated by other factors with a potential to increase inflammation in asthma, such as respiratory viruses or exposure to allergens. This inflammation creates, in itself, an increase in oxidative stress, which, in turn, activates AP-1 and NF-kb, factors that contribute to the perpetuation of inflammation [31,32].

## 2. The Role of Reactive Oxygen Species in the Pathogenesis of Bronchial Asthma

Humans have many complex systems: circulatory, respiratory, and neuroendocrine, which ensure constant oxygen levels [33]. The lungs are particularly vulnerable to oxidative stress, due to high ambient oxygen levels and exposure to pathogens and oxidants. ROS and reactive nitrogen species (RNS) can cause the oxidative destruction of DNA, lipids, carbohydrates, and proteins, contributing to the development and progression of pathology [34]. When accumulated, these events cause the formation of a vicious circle of persistent inflammation and chronic oxidative stress that causes an imbalance in the protease–anti-protease balance, as well as deficiency in tissue repair mechanisms, acceleration of apoptosis, and progression to chronic obstructive pulmonary disease (COPD) [35]. Oxidative stress has also been proven to be involved in decreased sensitivity to corticosteroids by altering the expression and signaling of the glucocorticoid receptor (GR) [36,37,38].

An atom contains a nucleus around which electrons usually move in pairs. Molecular oxygen is vital for energy and is essential for life. A free radical is any atom/molecule containing one or more unpaired electrons, which gives it high reactivity [39]. The body’s adaptive responses to the lack of oxygen do not take long to appear, being initiated by hypoxia-induced factor-1 (HIF-1). Hypoxia-induced factor-1 (HIF-1) mediates the adaptive response of cells to survive oxygen deprivation. One way in which cells survive oxygen deprivation is the transition from oxidative to glycolytic metabolism. Under aerobic conditions, electrons are transferred from NADH and flavin-adenine-nucleotide-2 (FADH2) to mitochondrial complex I and II, and then to complexes III and IV, where they react with oxygen to form water [40].

In mammals, ROS are produced by endogenous pro-oxidant enzymes such as NADPH-oxidase, xanthine-oxidase (XO), peroxisome enzymes, and cytochrome P-450 [41,42,43]. Patients with asthma exhibit oxidative stress and increased ROS, which suggests that their endogenous antioxidant mechanisms may be insufficient to prevent oxidative lesions. Under hypoxic conditions, the loss of redox homeostasis occurs due to the release of electrons, which lead to the production of superoxide anions, which are then converted into other ROS and hydrogen peroxide [44]. The loss of homeostasis at this level causes the generation of ROS and the activation of intracellular signals that become a triggering factor for the production of pro-inflammatory mediators, stimulating the development of histological changes at the lung level. The accumulation of highly reactive species increases lipid peroxidation and causes DNA destruction [45]. Therefore, ROS directly impact cell differentiation and proliferation, as well as the immune function and vasoregulation [46]. The cell protects itself from the damage caused by oxidative stress through an extensive network of enzymatic and non-enzymatic molecules, overall called antioxidants. Antioxidants work by maintaining a physiological balance between the generation of ROS and their removal [47,48]. Imbalances in the oxidant/antioxidant state are causally related to the pathophysiology of asthma, being involved in the development of airflow obstruction, hyperreactivity, and bronchial remodeling [49]. Apart from higher amounts of ROS synthesized in the lungs of asthmatic patients, antioxidative levels have been proven to be lower than those found in subjects with healthy lungs [50].

Although the epithelial barrier is well formed, children are more vulnerable to external insults in early life because of immune system immaturity [51]. Before the clinical diagnosis of asthma in preschool children, airway remodeling following exposure to environmental insults has been observed [52]. Prenatal and early postnatal exposure to passive cigarette smoke induces adverse effects on lung development and immune system, increasing risk of asthma occurrence [18,53]. Oxidative stress increases TLR4 expression and activation by bronchial epithelial cells [54], leading to prolonged pro-inflammatory responses. It also alters the barrier function acting on the airways’ remodeling and on the organization of their cytoskeleton [55].

## 3. Antioxidant Defense

The lungs are exposed to endogenous ROS, such as the final products of a cell’s metabolism (biochemical anabolic and catabolic reactions that occur in all living cells) [56], inflammatory cells (neutrophils, eosinophils, and macrophages) [57], fibroblasts, and epithelial cells and exogenous ROS (pollutants and cigarette smoke). At the cellular level, mitochondria are the main producers of ROS, with 1 to 3% of the electrons that reach their level forming a superoxide anion [58]. Firstly, for free radical formation, the activation of the enzyme complex of nicotinamide adenine dinucleotide reduced phosphate (NADPH) oxidase is needed. NADPH is present in the cell membrane and its activation produces the superoxide anion [59], which, in turn, is transformed to H_2_O_2_ and molecular oxygen (O_2_). Both O_2_^−^ and H_2_O_2_ can react, in the presence of iron or other metals, to form the most potent OH° radical [60].

In asthma pathophysiology, the main sources of ROS are inflammatory cells. The granulocytes peroxidases catalyze the reaction of H_2_O_2_, leading to the formation of hypochlorous acid (HClO) [61]. Eosinophils increase the ROS formation in the antigenic response of asthmatic patients [62]. Furthermore, leukocytes increase the production of superoxide anions, indicating that both intravascular inflammatory and pulmonary cells contribute to asthma oxidative stress [63].

The airway epithelium produces few antioxidants, which explains the inability to maintain the airway homeostasis [64]. To combat ROS formation, the enzymatic and non-enzymatic systems at the respiratory level are involved. The main enzyme systems are the SOD complex, which converts the superoxide anion into hydrogen peroxide; and glutathione peroxidase (GSH-Px) and reductase (GR), which inactivate hydrogen peroxide and other hydroperoxides and catalase, which, in turn, convert hydrogen peroxide into water and molecular oxygen [65]. SOD is highly expressed extracellularly around the airways and smooth muscles, and in the epithelium [66], counteracting the oxidative stress in asthma. It has been shown that asthmatic patients are deficient in antioxidant defenses, thus developing pulmonary dysfunction due to oxidative stress [67].

Nitric oxide (NO) is a molecule that regulates both bronchomotor tone and pulmonary vascular tone in the airways. In addition, NO participates in the host’s defense against infection through changes in barrier function, vascular permeability, repair, cytotoxicity regulation, ciliary motility, mucus secretion, and inflammatory cell infiltration [68]. With all these key functions, NO plays an important role in the pathogenesis of chronic inflammatory diseases of the airways [69]. NO has an inducible form (iNOS), which is regulated by proinflammatory stimuli, leading to the production of large amounts of NO for a long period of time [68,70].

NO plays an important role in reducing airway hyperresponsiveness (AHR) in the early phase of the asthmatic response; thus, a hyperreactive response is related to NO deficiency, either due to the lower activity of NOS constitutive isoforms or due to the greater activity of the enzyme arginase, which prevents the conversion to NO and L-citrulline by NOS [44]. In contrast, in the late phase of the asthmatic response, the increased activity of iNOS, which produces large amounts of NO, is responsible for AHR [71,72].

In asthma, inflammation and AHR are not linked to the production of NO itself, but are linked to the formation of the free radical, strong oxidizing agent, peroxynitrite, which results from the reaction of NO with the superoxide anion in the airways [73,74]. Peroxynitrite increases microvascular permeability, activates eosinophils, increases mucin 5AC (MUC5AC) expression, induces epithelial damage, and increases contraction of smooth muscles in the airways [75]. Peroxynitrite radical can also produce harmful effects on the respiratory system, leading to inflammatory damage, and can reduce the availability of NO [76].

**Figure 1:**
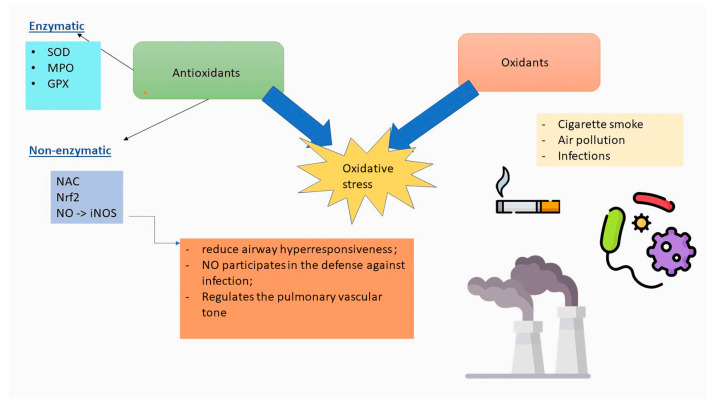
The main molecules involved in antioxidant defense: SOD—superoxide dismutase; MPO—myeloperoxidase; GPX—glutathione peroxidase; NAC—N-acetylcysteine; Nrf2—Erythroid 2 Nuclear factor; NO—nitric oxide; iNO—inducible form of NO.

Figure 1 illustrates the main molecules involved in antioxidant defense. Under physiological conditions, the level of oxidant species is dynamically stabilized by enzymatic and non-enzymatic cellular processes that produce or eliminate ROS.

### 3.1. Endogenous Enzymatic Antioxidants

Enzymatic antioxidants work by converting, in several steps, oxidizing metabolic products into hydrogen peroxide and then into water, using co-factors such as Fe, Zn, Cu, and Mn.

Myeloperoxidase (MPO) is an iron-containing enzyme with antimicrobial activity, being a component of azurophilic granules in neutrophils. Individuals with MPO deficiency may be at increased risk of inflammatory and infectious pathologies [77].

Superoxide dismutase (SOD) is an enzyme that can control the level of reactive oxygen species and nitrogen by catalyzing the conversion of superoxide to hydrogen peroxide and oxygen. It has three isoforms: cytoplasmic: Cu-ZnSOD (SOD1), mitochondrial MnSOD (SOD2), and extracellular EC-SOD (SOD3). The presence of specific SOD isoforms supports the importance of maintaining redox homeostasis among cellular compartments [78]. Changes in the SOD activity in a particular compartment could lead to the generation of a hydrogen peroxide concentration gradient and the activation of pathophysiological redox pathways. Low levels of this enzyme have been found in lung epithelial cells in patients with asthma, and the loss of enzyme activity can occur within minutes in response to acute airway damage. These changes contribute to an increase in ROS and oxidative stress levels, and ultimately, increased bronchial hyperresponsiveness and remodeling during asthmatic exacerbation [79,80,81].

Catalase is a heme-containing oxidoreductase located in peroxisomes. It works alongside glutathione peroxidase (GPX) and is responsible for converting hydrogen peroxide into O_2_ and water. Prolonged oxidative stress causes a decrease in its activity; therefore, its effectiveness is reduced in chronic pathologies such as bronchial asthma. Inactivation of this important antioxidative mechanism contributes to persistent airway inflammation and insensitivity to corticosteroid treatment [82,83].

Glutathione peroxidases (GPXs) make up a complex of eight isoforms, of which four are expressed at the lung level. They are involved in redox reactions alongside catalases, ultimately determining the physiological regulation of peroxide concentrations in intra- and extracellular compartments. Glutathione peroxidases (GPXs) use the reducing equivalents of GSH (the major non-enzymatic lung antioxidative glutathione) to reduce the level of cell-generated hydrogen peroxide in H_2_O. GPXs are much more versatile, functioning as both repair and ROS-removing enzymes [84]. Overall, GSH levels are lower in the serum and lung cells of children and adults diagnosed with asthma. The level of GSH is also low in exhaled air, but this level will increase with the initiation of oral corticosteroids [85,86].

Thioredoxin (TRX): TRX is another oxidoreductase with a protective role in the context of bronchial asthma [87]. It has two isoforms: TRX1, located mainly in the cytosol, and TRX2, located in the mitochondria and mostly maintained in the reduced form [88].

XO (xanthine oxidase): the level of this enzyme is higher in the sputum of patients with asthma than in healthy subjects. The use of XO inhibitors such as allopurinol or febuxostat may provide some benefit [89].

### 3.2. Endogenous Non-Enzymatic Antioxidants

Erythroid 2 Nuclear factor (Nrf2): Nrf2 is a transcription factor that regulates the expression of genes associated with protecting cells from oxidative stress and its damage. It contributes to the maintenance of lung homeostasis and toleration of oxidative-induced lung damage, and is important for slowing down the proinflammatory responses [90,91]. In severe asthma, Nrf2 activity and expression are significantly reduced [92,93]. Nrf2 activators can prevent autoimmunity-induced oxidative stress [94]. In studies on animals diagnosed with bronchial asthma, pharmacological activators of Nrf2 in lung epithelial cells represented a potential therapeutic target to reduce susceptibility to develop asthma [95]. They contribute to increasing the integrity of the bronchial epithelial barrier, by protecting the epithelial tight-junction proteins more than the use of inhaled steroids, to the inhibition of pulmonary inflammation, and to the improvement in sensitivity to corticosteroids [96,97,98,99,100,101,102].

N-acetylcysteine (NAC): NAC is a classic antioxidant that provides cysteine to increase intracellular glutathione production. In fact, NAC is a pleiotropic medicine with different pharmacological characteristics. It was developed as a mucolytic agent because it breaks down mucin disulfide bonds, reducing mucus viscosity and the level of pulmonary secretions and restoring blood oxygen saturation [99]. It has direct and indirect antioxidant properties. As a precursor of glutathione, NAC may exert an indirect antioxidant effect [100,101]. It has a free thiol group that is able to interact with the electrophilic groups of ROS, and it could be used as a protective factor against internal and external ROS [102]. In the HIACE (The Effect of High Dose N-acetylcysteine on Air Trapping and Airway Resistance of Chronic Obstructive Pulmonary Disease-a Double-blinded, Randomized, Placebo-controlled Trial) study [103], oral NAC administration (1200 mg/day for one year) showed significant improvement in functional tests for small airways and in the frequency of exacerbations. In studies carried out on animals diagnosed with corticosteroid-resistant asthma, the treatment with N-acetylcysteine (NAC) reduced airway inflammation to a greater extent than dexamethasone [104].

Glutathione is a low-molecular-weight tripeptide, abundant in most cellular compartments. It has the ability to regenerate other oxidized antioxidants, such as vitamin E and vitamin C, and is involved in the repair of peroxidized lipids [105]. Glutathione deficiency has been associated with asthma [106].

Human serum albumin (HSA) is the main extracellular protein that maintains plasma in the redox state [107]. It is known for its ability to bind various types of molecules; therefore, the antioxidant activity could result from binding to bilirubin, homocysteine, or lipids. For example, binding to polyunsaturated fatty acids could prevent lipid peroxidation [108].

## 4. Dietary Antioxidants and Their Role in Lung Pathology (Figure 2)

Vitamins C and E capture and remove the oxidation produced by RONS, especially at the level of membrane lipids [109]. Vitamin C has been shown to reduce oxidative stress, inflammation, and remodeling at the bronchial level [110,111]. Similarly, the vitamin E isoform gamma-tocotrienol has been proven to reduce house dust mite-induced allergic airway inflammation by inhibiting NF-kB and increasing Nrf2 activation [112]. The effects of gamma-tocotrienol on inflammation were comparable to those of corticosteroid treatment. Tocopherol supplementation has recently been demonstrated to reduce eosinophilic and neutrophilic inflammation in patients with asthma.

**Figure 2:**
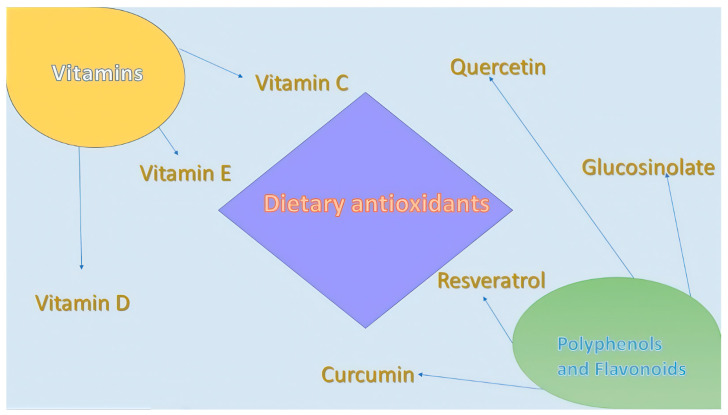
Dietary antioxidants.

Vitamin C provides adequate antioxidant capacity, both intra- and extracellularly. It removes a variety of free radicals and oxidative factors. In addition, ascorbate prevents lipid peroxidation through its reaction with membrane alpha-tocopherol [113].

Alpha-tocopherol (vitamin E) is present in low concentrations in the surfactant fluid and is thought to be secreted by alveolar type II cells. It is a powerful antioxidant due to its ability to remove free radicals and stop lipid peroxidation reactions [114]. Vitamin E protects polyunsaturated fatty acids in the cell membrane against lipid peroxidation, thus preserving cell membrane functions. It was discovered that patients with asthma have reduced concentrations of vitamin E and vitamin C in the pulmonary surfactant, thus being more affected by oxidative stress. Vitamin E supplementation has a positive effect on the development of immunity, fetal lung function, and the reduction in the wheezing incidence [115].

A randomized study [116] conducted on children diagnosed with bronchial asthma, who were citizens of Mexico, aimed to highlight the effects of vitamin E and C administration in patients exposed to ozone. In vivo studies on animals have shown that vitamin E supplementation has a protective effect against lipid peroxidation and that the level of vitamin E in the lung tissues significantly increased after the animals’ exposure to ozone. This observation suggests that in response to oxidative stress, vitamin E is mobilized towards the lung tissue in response to oxidative stress. The children in the study mentioned above randomly received either daily vitamin supplements of vitamin E = 50 mg/day or vitamin C = 250 mg/day, or placebo, and were monitored from October 1998 to April 2000. Significant differences in lung function were observed between groups for FEF25-75 and PEF in patients with moderate-severe asthma. The results of this study suggest that antioxidant supplementation could modulate the impact of ozone exposure on the small airways of children with moderate-severe asthma. The protective effect of this supplementation appears to be greater in patients with moderate asthma than in those with mild asthma.

Zinc (Zn) has an antioxidant effect by reducing the formation of the hydroxyl radical from hydrogen peroxide. Moreover, Zn acts as a co-factor for SOD. Several observational studies have demonstrated that asthmatic patients have lower serum levels of Zn, vitamin C, and vitamin E than the healthy population [117,118].

A prospective cohort study carried out in the United Kingdom [119] confirmed that a high dietary intake of foods rich in vitamin C or vitamin C itself slowed the decline in lung function. Similarly, another study showed that an increased intake of fruits, especially tomatoes, a rich source of lycopene-carotenoid, also resulted in a delay in the decline in lung function [120].

Broccoli is known to have an increased content of bioactive phytochemicals such as glucosinolate, phenolic compounds, vitamin C, and minerals [121]. For example, sulforaphane extract, a sulfur-rich component found in this vegetable, has been documented to increase Nrf2 and its antioxidant properties in the superior respiratory airways, as well as to improve the ability of macrophages to phagocytose bacteria [122,123]. On the other hand, a cross-sectional study [83] based on a Korean population showed that subjects with the lowest intake of vitamin A, vitamin C, and beta-carotene had remarkably lower forced expiratory volume per second (FEV1) values than those with a higher intake.

In another cross-sectional study carried out on the general population, a 20 mmol/L increase in plasma vitamin C concentrations caused a 13% decrease in the risk of chronic lung disease [124]. Moreover, resveratrol, a constituent flavonoid in red wine, has been studied for its properties to reduce GSH synthesis and inhibit the release of inflammatory cytokines [125]. Another polyphenol, curcumin, has been found to inhibit the inflammatory response by suppressing NF-κB activity and recruit neutrophils at the lung level [126].

Although through different mechanisms, vitamin D has been prescribed to some asthmatic patients. An important immunoregulatory and anti-inflammatory cytokine is IL-10, which is secreted by T cells in response to glucocorticoids [127]. Glucocorticoid-resistant asthmatics do not have enough secretion of IL-10, and when they receive an intake of D3, this protection through interleukins is restored, which suggests the possibility of involvement of vitamin D3 in therapy.

Fruits and vegetables are also rich in polyphenols and flavonoids, and have antioxidant activity. Table 1 summarizes polyphenols that play an important role in protection against asthma. Substances such as quercetin and resveratrol have been shown to increase endogenous activation of Nrf2 while reducing pulmonary inflammation and oxidative stress [128,129,130]. In airway epithelial cells, curcumin has been shown to improve corticosteroid sensitivity by increasing the expression of the HDAC2 receptor [131]. Flavonoids have stronger antioxidant effects compared to vitamin C and vitamin E [132]. The antioxidant properties depend on their chemical structure, especially on their catechol group. The more that flavonoids contain substituted hydroxyl groups, the more antioxidative activity they have. They have strong antioxidant activity and the ability to regulate intracellular signaling pathways [133].

Kaempferol, a flavonoid found in apples and many berries, has been shown to reduce airway inflammation and act as a therapeutic agent for asthmatics. In bronchial epithelial cells, it inhibits eotaxin-1 and LPS-induced IL-8 production by activating TLR4 [134]. Kaempferol’s effect on NF-kB signaling was further confirmed in subjects with TNFα-induced lung inflammation [135].

Another widely studied flavonoid, quercetin, found in vegetables, fruits, nuts, tea, and wine, is known for its strong effects on ROS metabolism and cell apoptosis. Quercetin supplementation showed marked reductions in malondialdehyde, TNFα/IL-10, and IL-8/IL-10, markers of oxidative stress. It reduces the production of pro-inflammatory cytokines by modulating pathophysiological pathways driven by NF-kb1 and I kb [136].

Isorhamnetin, the metabolite of quercetin, is a flavonol found in turnip leaves, almonds, red onion, and fennel, and has been described for its anti-proliferative, antioxidative, and anti- inflammatory effects. It has been shown to antagonize H_2_O_2_ production and reduce apoptotic destruction by inhibiting cytochrome C release [137].

Dietary antioxidants have been explored for their ability to influence lung function. The association between the dietary total antioxidative capacity (TAC) of 8-year-old children, and their lung function at 16, was analyzed in a cohort study carried out in Sweden. Their lung function was measured at the ages of 8 and 16 by impulse oscillometry, spirometry, and measurement of exhaled nitric oxide. For the total population, no statistically significant associations were observed between TAC and spirometry results, but among children diagnosed with asthma at the age of 8, a diet with more TAC was associated with higher average FEV1 values [138,139]. The analysis from the Swedish BAMSE [134] cohort also shows that high dietary antioxidative intake at the age of 8 was associated with a reduced risk of later IgE-mediated sensitization to inhaled allergens and allergic asthma. The TAC intake in this study is in line with actual recommendations for the general population, which urge the consumption of five servings of vegetables and fruits per day [140].

A slower decline in lung function 10 years after following a diet high in antioxidants from tomatoes and fruits was documented in many cross-sectional and prospective studies [119,141,142,143]. In Puerto Rican children, a case–control study [141] showed that a diet low in sweets and diary and rich in grains and vegetables was linked to better pulmonary function, as measured by forced vital capacity (FVC) and forced expiratory volume in 1 s (FEV1). However, the response to dietary antioxidants may be altered according to the environmental sources of oxidative stress, life stage, and genetic susceptibility. Furthermore, changes in the gut microbiome have been associated with lung diseases and altered immune responses [144,145].

Javid A. et al. examined 42 children with intermittent asthma at the onset of common cold symptoms. They randomly received either an herbal mixture (comprised of *Matricaria chamomilla*, *Althaea officinalis*, *Malva sylvestris*, *Hyssopus officinalis*, *Adiantum capillus-veneris*, *Glycyrrhiza glabra*, and *Ziziphus jujube*) or placebo for 5 days. The herbal mixture significantly decreased the severity of coughs and nighttime awakenings. There was no reduction in wheezing, tachypnea, peak end-flow rate (PEFR), asthma exacerbation, oral prednisolone, or β-agonist usage [146].

A multicenter, randomized study was conducted of 386 adults and children aged 12 years or older while taking a controller medicine. Participants were randomly divided in two groups: one in which patients received soy isoflavone supplement with 100 mg isoflavones for 24 weeks, and a placebo group. They concluded that no differences in improved lung function or clinical outcomes between the two groups were found. These findings suggest that this supplement should not be used in poorly controlled asthma [147].

Another study examined Chinese herbal medicine (CHM) for its capacity to relieve asthma symptoms and reduce airway hyperresponsiveness. Patients aged less than 18 years old and diagnosed with asthma were included in this study. The authors concluded that children aged 6–18 years who used CHM therapy for more than 6 months had a reduced risk of hospitalization, and long-term CHM therapy has a benefit in school-age children with asthma [148].

Research indicates that antioxidant responses may vary based on factors such as life stage, genetic predisposition, and environmental oxidative stress. A meta-analysis of 26 case–control studies involving 3000 participants linked deletions in GSTM1 and GSTT1 genes to an increased risk of asthma [149]. Genetic variations across ethnic groups contributed to study differences. For example, the absence of the GSTM1 gene was associated with asthma in Europe, Africa, and Latin America, while GSTT1 deletion was linked to a higher asthma risk in Asian and Russian populations. Another study by Romieu et al. [150] examined the influence of antioxidant supplementation on lung function in asthmatic children exposed to ozone, particularly in those with the GSTM1-null genotype. The results showed that children lacking the GSTM1 gene had more significant reductions in lung function after ozone exposure, but they responded better to antioxidant supplementation compared to GSTM1-positive children. These findings show how genetic factors, particularly GSTM1 and GSTT1 deletions, and environmental factors influence asthma susceptibility and responses to oxidative stress and antioxidants, with ethnic variability playing a role in these effects.

## 5. New Antioxidant Therapy

New options of antioxidant therapy are currently under investigation: selective NOX inhibitors [151]; Nrf2 agonists such as sulforaphane, which can initiate the activation of anti-inflammatory signaling [30]; N-acetylcysteine, proven to reduce air-pollution-mediated bronchial hyperresponsiveness [54,152]; coenzyme Q, a component of the mitochondrial electron transport chain capable of decreasing mitochondrial redox dysfunction; superoxide dismutase mimicking molecules [19,152,153]; selective and not-selective phosphoinositide 3-kinase (PI3Kδ) inhibitors; and inhaled p38 mitogen-activated protein kinases (p38MAPK) inhibitors. Among these molecules, some have the ability to activate HDAC2, inducing the transcription of anti-inflammatory genes and restoration of proper steroid sensitivity. It is shown that macrolides can be used as PI3Kδ inhibitors more distally in the molecular pathway thanks to their anti-inflammatory properties [154,155]. Furthermore, nanotechnology organelle-targeted therapies are currently under investigation for their strategical capacity to reach specific subcellular sites [156]. In a cellular model with high oxidative stress and corticosteroid resistance, nano- and micro-structured lipid carriers can be considered a new strategy to improve corticosteroid effects [157,158,159]. These therapies are currently under investigation and more research has to be conducted to establish their clinical utility.

Three in vitro studies [160,161,162] investigated various compounds and extracts for their potential as anti-asthma agents. The first study [160] focused on Chromenol (Mojobanchromanol) from *Sargassum horneri*, showing it reduced oxidative stress, inhibited MAPK signaling pathways, and suppressed pro-inflammatory cytokine secretion. The second study [161] explored Fucosterol from *Padina boryana*, which demonstrated anti-inflammatory effects by inhibiting MAPK and activating Nrf2/HO-1 pathways. The third study [162] investigated Fucoidan from *Saccharina japonica*, which effectively reduced nitric oxide (NO) and pro-inflammatory cytokines, blocking key inflammatory pathways like MAPK and NF-κB, and thereby showcasing its anti-inflammatory potential.

## 6. ROS Effect on the Therapeutic Response to the Administration of Corticosteroids

The current therapeutic protocol for pediatric patients diagnosed with bronchial asthma is based on two strategies: initial treatment and treatment guided by the evolution of the disease. The therapy is initiated according to the presence of exacerbations and physical limitations, as well as the frequency of symptoms during the day/night. Following the evaluation of the mentioned parameters and the institution of therapy, asthma can be classified as controlled, partially controlled, or uncontrolled [163,164]. Some xanthine derivative drugs can be used as an adjuvant therapy in acute asthma. Theophylline is a methylxanthine molecule with bronchodilator, anti-inflammatory, and immunomodulatory activities. It is used in persistent pediatric asthma where low-dose inhaled steroids are insufficient to control symptoms [165].

A theophylline derivate, doxofylline, is known to favor IL-10 release by phosphodiesterase inhibition. It has anti-inflammatory effects by linking to β2-adrenoireceptors, causing relaxation of blood vessel and bronchial smooth muscles [166,167].

Some studies were conducted in pediatric patients to demonstrate the clinical benefit of doxofylline administration. In the first, 5 mL/kg of doxofylline was administered to 116 children with acute asthma and the authors concluded that its use in asthma attacks is limited [168]. A double-blind study was conducted to evaluate the efficacy of doxofylline administered to children aged 6–12 years at the dose of 6 mg/kg every 12 h for 2 weeks. Significant differences for forced expiratory volume 1 (FEV1) and peak expiratory flow rate (PEFR) were found in the treated vs. placebo groups [169]. New research in this field has been conducted, and recent studies [169,170,171] show the effectiveness of doxofylline in asthma with an efficacy/safety profile better than that of theophylline. In conclusion, we can use a methylxanthine in addition to corticosteroid therapy in acute asthma, but more research in pediatric populations has to be performed. Kondo et al. randomly treated 6–14-year-old patients receiving inhaled corticosteroids (ICSs) with either montelukast 5 mg/day or theophylline 5–8 mg/kg, for 4 weeks. A remarkable increase in morning PEF was observed in the montelukast-treated group compared with the theophylline group. The authors concluded that montelukast added to low-dose ICS can be an effective and safe option in asthma treatment for children [172].

Inhaled corticosteroids in association with inhaled β2-agonists are now the standard treatment for asthma [173]. Corticosteroids have an effect on leukocyte recruitment, as well as anti-inflammatory effects by modulating the expression of AP-1 and NF-kB [174,175]. Although corticosteroid therapy is considered the gold standard, numerous studies have reported a significant incidence of adverse effects if used in high doses and in the long run [176,177,178].

The β2-adrenergic receptor, a receptor protein (expressed on many types of bronchial cells) linked to G protein encoded by the ADRβ2 gene, can be bound by the action of β2-adrenergic receptor agonists. This gene is highly polymorphic and influences lung function, as well as the response to β-agonist treatment [179,180,181].

The glucocorticoid receptor (GR) is internalized in the cytosol by corticosteroids, thus reducing inflammation. Corticosteroids can also activate GR translocation to the nucleus, where it will regulate gene expression [37,182]. In pathological situations, GR causes the impairment of genes and can activate the pathophysiological pathways associated with metabolic and inflammatory processes. Currently, GR is studied for its ability to suppress inflammation, suppress promoter activation in the pro-inflammatory genes, increase the expression of anti-inflammatory mediators, and modulate chromatin structure [183,184]. The anti-inflammatory effects of corticosteroids are due to their ability to modulate gene expression in multiple cell types, including epithelial cells, immune cells, smooth muscle cells, and fibroblasts. Recent studies [185,186,187] showed that GR effects are exerted differently depending on the cell type, which can be an important factor that may influence corticosteroid sensitivity in asthmatic patients.

Systemic and local glucocorticoid treatments are proven to not be effective in some patients with severe pulmonary damage exposed to pro-oxidative agents [188,189,190,191]. Glucocorticoid resistance is defined, in clinical terms, as a failure to increase the forced expiratory volume in the first second (FEV1) by 15% after 7 days of treatment with oral corticosteroids [192].

The development of corticosteroid resistance is linked to several mechanisms, as follows: Immune-mediated resistance occurs through the dysregulation of cytokines driven by the over-activation of mitogen-activated protein kinase (MAPK), nuclear factor kappa B (NF-κB), and activator peptide-1 (AP-1), resulting in deficiencies in the ability of GR to bind the medicine and translocate it to the nucleus. A key role in steroid resistance appears to be played by the Th17-mediated immune response, which determines the accumulation of neutrophils in inflammatory lung diseases [193]. Therefore, the inhibition of neutrophilic inflammation alone might be insufficient for steroid-resistant conditions because TNFa neutralization after high-dose corticosteroid treatment will not increase FEV1 levels in patients with severe asthma. GR inactivation decreases the ability of the receptor to induce histone acetylation, which in turn prevents the interaction with the pro-inflammatory transcription factors Aβ-1 and NF-κB [194].

Finally, an expression of an isoform of GR, GRβ, occurs in inflammatory cells. Although the β isoform of GR differs from the isoform by only one carboxyl terminal group, this difference is sufficient to avoid glucocorticoid binding. However, GRβ is able to bind to the glucocorticoid response element, even in the absence of the ligand, but cannot activate the promoter of glucocorticoid-responsive genes [195].

## 7. Conclusions

Bronchial asthma is a vast pathology that is still subject to the attention of researchers due to its complex pathophysiology, as well as its potential to introduce new therapeutic indications. A new therapeutic strategy in asthma could be represented by targeting the oxidative molecules. Considering that the formation of ROS, RNS, and lipid peroxidation products can lead to the formation and, later, to the progression of asthma, the use of antioxidants could have an ideal effect in the treatment of this pathology.

Dietary antioxidants such as vitamin C and E, minerals such as zinc, and, especially, flavonol compounds have been proven to be effective in numerous studies carried out in the pediatric population and could become therapeutic molecules for lung inflammatory diseases, not only because of their properties of internalizing the nitrogen and oxygen radicals, but also due to modulatory effects on cellular signaling pathways related to inflammation and lung pathology. An increased intake of antioxidants in the diet in early childhood (five servings of fruits and vegetables according to current WHO recommendations) has been shown to prevent the decline in lung function later in adolescence.

Although the therapeutic use of antioxidants in allergic asthma has not yet been recommended by GINA, improving airway redox balance could enhance corticosteroid sensitivity. Advances in the understanding of antioxidant mechanisms and their pharmacological targeting may provide new therapeutic opportunities, thus reducing the need for high-dose corticosteroids while preserving general anti-inflammatory effects.

## Figures and Tables

**Table 1:** Polyphenols that play an important role in protection against asthma and their dietary sources.

Dietary Polyphenols	Sources
Resveratrol	Grapes Blueberries Cranberries Peanuts Dark chocolate Pistachios Red wine Cocoa
Quercetin	Tea Onions Grapes Blueberries Blackberries Cocoa Citrus Green leafy vegetables Red cherries Tomatoes Apples
Curcumin	Rhizome Turmeric
Kaempferol	Strawberry Gooseberry red Gooseberry yellow Onion leaves Black tea Green chili Pumpkin Carrot White radish Beans Broccoli Cauliflower
Isorhamnetin	Apple Pears Red cherries Turnip greens Almonds Fennel leaves Chives Dill weed

## Data Availability

No new data were generated.
